# Do pre- and post-copulatory sexually selected traits covary in large herbivores?

**DOI:** 10.1186/1471-2148-14-79

**Published:** 2014-04-10

**Authors:** Mariona Ferrandiz-Rovira, Jean-François Lemaître, Sophie Lardy, Bernat C López, Aurélie Cohas

**Affiliations:** 1 Laboratoire Biométrie et Biologie Evolutive, Université de Lyon, CNRS, UMR5558, Université Lyon 1, F-69622, Villeurbanne, F-69000 Lyon, France; 2 Groupe Ecologie et conservation des vertébrés, Université d’Angers, Faculté des Sciences, 49045 Angers, France; 3 CREAF, Cerdanyola del Vallès, 08193 Catalunya, Spain; 4 Universitat Autònoma de Barcelona, Cerdanyola del Vallès, 08193 Catalunya, Spain

**Keywords:** Pre-copulatory competition, Post-copulatory competition, Secondary sexual traits, Sexual selection, Sperm competition, Weapon length

## Abstract

**Background:**

In most species, males compete to gain both matings (via pre-copulatory competition) and fertilizations (via post-copulatory competition) to maximize their reproductive success. However, the quantity of resources devoted to sexual traits is finite, and so males are predicted to balance their investment between pre- and post-copulatory expenditure depending on the expected pay-offs that should vary according to mating tactics. In *Artiodactyla* species, males can invest in weapons such as horns or antlers to increase their mating gains or in testes mass/sperm dimensions to increase their fertilization efficiency. Moreover, it has been suggested that in these species, males with territory defence mating tactic might preferentially increase their investment in post-copulatory traits to increase their fertilization efficiency whereas males with female defence mating tactic might increase their investment in pre-copulatory sexually selected traits to prevent other males from copulating with females. In this study, we thus test the prediction that male’s weapon length (pre-copulatory trait) covaries negatively with relative testes size and/or sperm dimensions (post-copulatory traits) across *Artiodactyla* using a phylogenetically controlled framework.

**Results:**

Surprisingly no association between weapon length and testes mass is found but a negative association between weapon length and sperm length is evidenced. In addition, neither pre- nor post-copulatory traits were found to be affected by male mating tactics.

**Conclusions:**

We propose several hypotheses that could explain why male ungulates may not balance their reproductive investment between pre- and post-copulatory traits.

## Background

Understanding the diversity and evolution of male sexual traits is a major interest in evolutionary ecology [1,2], and there is now substantial evidence that sexual competition has shaped these traits [1,3]. Firstly, males compete for mating opportunities (i.e. pre-copulatory competition) and individuals who invest in secondary sexual traits such as ornaments or weapons are often better competitors and gain more mating events (e.g. [4,5]). Secondly, in species where females mate with more than one male in the same reproductive bout, males compete to fertilize a set of ova through sperm competition (i.e. post-copulatory competition) [3]. In many cases, sperm competition can be compared to a raffle where the male probability to fertilize eggs is proportional to the relative number of sperm delivered by the male [6,7]. Sperm traits such as sperm dimensions and characteristics can also increase the probability of fertilization through their impact on sperm mobility and viability [8]. Such sperm characteristics can be considered as sperm quality characteristics and can also play an important role in the outcome of sperm competition [7,8]. Consequently, sperm competition has selected for adaptations that increase both sperm quantity and/or quality. For example, in mammalian taxa, testes mass (relative to body mass), a proxy of sperm quantity [9], is strongly associated with different proxies of sperm competition such as social group size in bats [10] or the percentage of within-litter multiple paternity in rodents [11]. Moreover, although sperm quantity has been traditionally emphasized, recent studies testing simultaneously the contribution of both sperm quantity and quality on fertility have shown that, under some circumstances, sperm quality could be more important than quantity. For example, one study found that in the white-footed mouse (*Peromyscus leucopus noveboracensis*), the probability of siring litters was more affected by sperm quality (measured as sperm viability and percentage of sperm presenting abnormalities) than sperm quantity [12].

The expression and maintenance of sexual traits involved in both pre- and post-copulatory competition are energy and time consuming (e.g. [13,14]) and are generally associated with various costs. For example, conspicuous secondary sexual traits often cause high predation risks [15] and male investment in both pre- and post-copulatory traits decreases immune efficiency [16,17]. Since males can allocate only a limited amount of resources to sexual competition [6], theoretical models of sperm competition predict that, when the relative intensity of pre- or post-copulatory pressures change, males should modulate their reproductive investment between traits involved in competition for mating and traits involved in the production of high quality ejaculates depending on the expected pay-offs [6,18]. Basically, when females are likely to mate with different males (i.e. high level of sperm competition), males should preferentially increase their investment in ejaculate expenditure by decreasing the investment to pre-copulatory male sexually selected traits; whereas when female propensity to mate multiply is weak (i.e. low level of sperm competition), males should predominantly increase their investment to pre-copulatory male sexually selected traits by decreasing the investment in ejaculate expenditure [6,18]. So far, evidence for such relationships at the inter-specific level comes mainly from two studies: (1) Simmons and Emlen [19], who found in beetles from the genus *Onthophagus,* that species with the steepest allometric slopes of horn size on body size also display the shallowest allometric slopes of testes mass on body size (and vice versa), and (2) Fitzpatrick et al. [20], who recently revealed a negative relationship between sexual size dimorphism and both baculum length and testes mass across pinniped species. Although male development and maintenance of pre-copulatory male sexually selected traits other than body size or mass are costly (i.e. [21]), and can potentially covary with the expression of ejaculate characteristics (i.e. [22]), inter-specific studies involving male armaments or ornamental traits remain scarce. Moreover, in this context, investment in sperm quality has never been considered, maybe because models of allocation to sperm competition according to the level of pre-copulatory competition are principally focused on sperm quantity [6,18].

Ungulates are well-suited to examine covariation between investment in sexual traits since males in this group face intense competition both to secure matings and fertilize ova and developed traits to increase their success under both pre- and post-copulatory competition [23,24]. The predominant mating system of ungulate species is polygynous [25] and conspicuous weapons such as horns for *Bovidae* and antlers for *Cervidae* have been sexually selected in this group because they provide an advantage for males in gaining matings and ultimately increase fitness [23]. For example, in Soay sheep (*Ovis aries*) larger horns enhance the probability of being observed in consort, which in turn is related to mating success [4]. In addition, ungulate species where males face a high level of sperm competition have developed larger testes [26,27], and sperm velocity is also likely to be under strong selective pressure since this parameter is directly related to variation in male fertility (e.g. red deer in Malo et al. [28]). Moreover, it has been hypothesized that the level of intra-male competition and ultimately the relative investment in both pre- and post-copulatory traits could potentially differ between mating tactics exhibited by males in polygynous ungulates [23]. In the female defence mating tactic, males follow females and attempt to guard them during oestrus. Therefore, male mating opportunities might depend predominantly on their dominance hierarchy and abilities to prevent other males from copulating with females mainly from the use of their weapons [24]. On the contrary, in the territory (or resource) defence tactic, males defend areas to attract females but do not monopolize females that can freely range over several male territories. As a consequence, the risk of sperm competition might be higher in species with territory defence tactic and males should increase their investment in ejaculate expenditure as predicted by a recent model of sperm competition [18]. Thus, the variation found in ungulate sexual traits (weapon length in Bro-Jørgensen [23] and Plard et al. [29] and both testes mass and sperm length in Gomendio et al. [30]) could ultimately be the result of different levels of sperm competition between mating tactics exhibited by males.

In this study, we first use a comparative analysis on ungulates to test for covariation between male investment in weapon (horns or antlers) length and male investment in sperm quantity and/or quality (relative testes mass and/or sperm dimensions). We expect that the relative size of these pre- and post-copulatory traits should be negatively correlated [18]. Then we tested the hypothesis that male mating tactic (female or territory defence) can mediate the relative investment in these pre- and post-copulatory traits. Following Bro-Jørgensen [23], we expect species with a female defence mating tactic to invest in pre-copulatory male sexually selected traits at the expense of their investment in post-copulatory traits, while we expect the reverse pattern for species with a territory defence mating tactic.

## Results

Regarding post-copulatory traits, no significant relationships between relative testes mass and sperm dimensions were observed (Additional file 1: Table S1a). However, positive relationships between sperm dimensions were evidenced (see Additional file 1: Table S1b and S1c).

**Figure 1:**
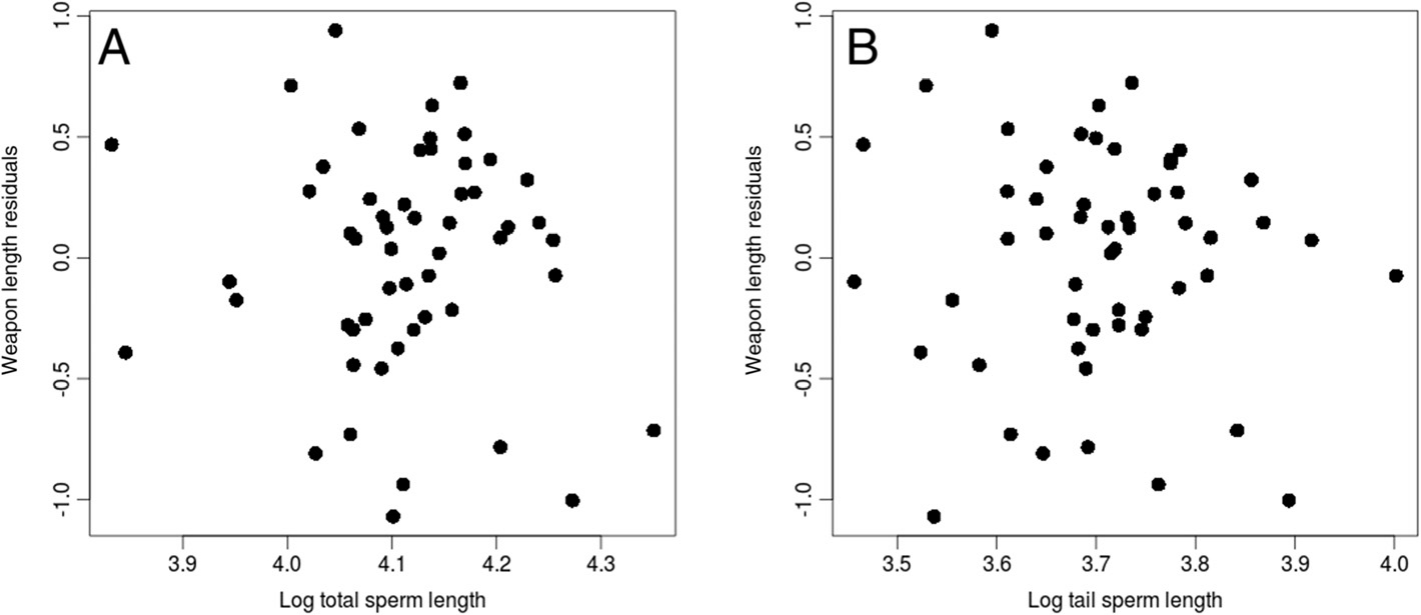
**Weapon length (after correction for body mass allometry) in relation to total sperm length (a) and tail length (b) without considering phylogeny for 54 species (both *Bovidae *** **and *Cervidae *** **pooled).**

No significant relationship between relative weapon length and relative testes mass was found (Table 1). However, there was evidence for a strong significant negative relationship (r = −0.27) between weapon and total sperm length (Figure 1a, Table 1). Although lengths of all sperm components (head, midpiece and tail) are significantly longer as total sperm length increases (see Additional file 1: Table S1b and S1c), only tail length was found to be strongly negatively associated with weapon length (r = − 0.30) although this relation was found to be significant only with the phylogenetic tree derived from Bininda-Emonds et al. [31] (p = 0.03 with Bininda-Emonds et al. [31]; r = − 0.26, p = 0.07 with Agnarsson and May-Collado [32] tree) (Figure 1b, Table 1, Additional file 2: Table S2a). No significant associations were found between weapon length and head or midpiece length, nor between weapon length and midpiece volume (Table 1). The taxonomic family had no effect on the evidenced relationships (see Additional file 3: Table S3).

**Table 1:** Phylogenetically corrected models testing the relationships between male pre- and post-copulatory sexually selected traits

**Dependent variable**	**Independent variables**	**Beta ± SE**	**t**	**P**	**N**	**df**	**r**	**λ**
Weapon length	Body mass	0.48 ± 0.09	5.58	< 0.001	45	42	0.65	0.69^**0.36/0.21**^
	Testes mass	0.04 ± 0.08	0.50	0.62			0.08	
Weapon length	Body mass	0.56 ± 0.06	10.01	< 0.001	54	51	0.81	1.00^**< 0.001/1**^
	Sperm length	−0.95 ± 0.47	−2.04	0.05			−0.27	
Weapon length	Body mass	0.58 ± 0.06	10.43	< 0.001	54	51	0.83	0.98^**0.01/< 0.001**^
	Sperm head length	−0.04 ± 0.29	−0.12	0.90			−0.02	
Weapon length	Body mass	0.56 ± 0.06	9.10	< 0.001	53	50	0.79	0.98^**< 0.001/0.26**^
	Sperm midpiece length	−0.11 ± 0.31	−0.35	0.73			−0.05	
Weapon length	Body mass	0.51 ± 0.06	8.37	< 0.001	47	44	0.78	0.98^**< 0.001/0.21**^
	Sperm midpiece volume	0.02 ± 0.07	0.35	0.73			0.05	
Weapon length	Body mass	0.57 ± 0.05	10.58	< 0.001	54	51	0.83	1.00^**< 0.001/1**^
	Sperm tail length	−0.80 ± 0.36	−2.24	0.03			−0.30	

Models are tested with Bininda-Emonds et al. [31] phylogeny across ungulates species (both *Bovidae* and *Cervidae* pooled). r represents effect size values. λ represents the index of phylogenetic covariance (see Methods section). The superscripts following the λ value indicate p-value of likelihood ratio tests against models with λ = 0 (first position) and λ = 1 (second position). All variables were log transformed.

No relationships between mating tactics and any of the pre- and post-copulatory traits were evidenced (Table 2). Furthermore, the mating tactics did not modulate the relationships between weapons length and the different post-copulatory traits (Table 3).

**Table 2:** Phylogenetically corrected models testing for differences in male pre- and post-copulatory sexually selected traits with different mating tactics

**Dependent variables**	**Independent variables**	**Beta ± SE**	**t**	**P**	**N**	**df**	**r**	**λ**
Weapon length*	Body mass*	0.53 ± 0.07	7.67	< 0.001	58	55	0.72	0.98^**0.001/0.30**^
	Tactic	−0.22 ± 0.17	−1.29	0.20			−0.17	
Testes mass*	Body mass*	0.67 ± 0.12	5.51	< 0.001	45	42	0.65	0.00^**1/< 0.001**^
	Tactic	0.16 ± 0.27	0.60	0.55			0.09	
Sperm length	Tactic	−1.02 ± 1.94	−0.53	0.60	54	52	−0.07	0.67^**0.06/< 0.001**^
Sperm head length	Tactic	−0.16 ± 0.41	−0.38	0.70	54	52	−0.05	0.58^**0.01/< 0.001**^
Sperm midpiece length	Tactic	0.04 ± 0.78	0.05	0.96	53	51	0.01	0.92^**< 0.001/0.01**^
Sperm midpiece volume	Tactic	−0.13 ± 0.39	−0.34	0.74	47	45	−0.05	0.00^**1/0**^
Sperm tail length	Tactic	−1.23 ± 1.50	−0.82	0.42	54	52	−0.11	0.52^**0.24/< 0.001**^

Female defence species is coded as 0 and territory defence is coded as 1. Models are tested with Bininda-Emonds et al. [31] phylogeny across ungulates species (both *Bovidae* and *Cervidae* pooled). r represents effect size values and λ represents the index of phylogenetic covariance (see Methods section). The superscripts following the λ value indicate p-value of likelihood ratio tests against models with λ = 0 (first position) and λ = 1 (second position). *log transformed variables.

**Table 3:** Phylogenetically corrected models testing the relationship between weapon length and testes mass, sperm dimensions and mating tactics

**Dependent variable**	**Independent variables**	**beta ± SE**	**t**	**P**	**N**	**df**	**r**	**λ**
Weapon length	Body mass	0.44 ± 0.09	4.70	< 0.001	45	41	0.59	0.27^**0.64/0.11**^
	Testes mass	−0.04 ± 0.12	−0.31	0.76			−0.05	
	Tactic	−0.74 ± 0.60	−1.22	0.23			−0.19	
	Testes mass × Tactic	0.11 ± 0.14	0.74	0.46			0.12	
Weapon length	Body mass	0.56 ± 0.06	8.40	< 0.001	54	50	0.77	1.00^**< 0.001/0.81**^
	Sperm length	−1.92 ± 1.05	−1.83	0.07			0.25	
	Tactic	−5.12 ± 4.74	−1.08	0.28			0.15	
	Sperm length × Tactic	1.20 ± 1.16	1.04	0.30			0.15	
Weapon length	Body mass	0.53 ± 0.06	8.53	< 0.001	54	50	0.77	0.99^**< 0.001/0.38**^
	Sperm head length	0.90 ± 0.76	1.18	0.24			0.16	
	Tactic	2.14 ± 1.69	1.26	0.21			0.18	
	Sperm head length × Tactic	−1.11 ± 0.81	−1.37	0.18			−0.19	
Weapon length	Body mass	0.52 ± 0.07	7.50	< 0.001	53	49	0.73	0.98^**< 0.001/0.36**^
	Sperm midpiece length	−0.26 ± 0.46	−0.56	0.58			0.08	
	Tactic	−0.59 ± 1.46	−0.41	0.69			−0.06	
	Sperm midpiece length × Tactic	0.16 ± 0.58	0.28	0.78			0.04	
Weapon length	Body mass	0.49 ± 0.07	7.26	< 0.001	47	43	0.74	0.98^**< 0.001/0.20**^
	Sperm midpiece volume	0.08 ± 0.17	0.45	0.65			0.07	
	Tactic	−0.07 ± 0.23	−0.28	0.78			−0.04	
	Sperm midpiece volume × Tactic	−0.06 ± 0.19	−0.34	0.74			−0.05	
Weapon length	Body mass	0.55 ± 0.06	8.91	< 0.001	54	50	0.78	1.00^**< 0.001/1**^
	Sperm tail length	−1.44 ± 0.75	−1.92	0.06			−0.26	
	Tactic	−3.06 ± 3.14	−0.98	0.33			−0.14	
	Sperm tail length × tactic	0.78 ± 0.85	0.91	0.37			0.13	

Female defence species is coded as 0 and territory defence is coded as 1. Models are tested with Bininda-Emonds et al. [31] phylogeny across ungulates species (both *Bovidae* and *Cervidae* pooled). r represents effect size values and λ represents the index of phylogenetic covariance (see Methods section). The superscripts following the λ value indicate p-value of likelihood ratio tests against models with λ = 0 (first position) and λ = 1 (second position). All variables were log transformed.

## Discussion

Surprisingly, no evidence of covariation between relative weapon length and relative testes mass was found across ungulate species. However our results show evidence of a negative association between relative weapon length and sperm length. Moreover, we found that allocation to pre- and post-reproductive traits is independent of the mating tactic.

The absence of a negative covariation between relative weapon length and relative testes mass contrasts with both theoretical predictions [18] and recent empirical findings from Fitzpatrick et al. [20], who found a negative relationship between sexual size dimorphism and relative testes mass across thirteen species of pinnipeds. One possible explanation for the apparent discrepancy between these two studies could come from a much lower intensity of post-copulatory competition in ungulates compared to pinnipeds as suggested by their small relative testes mass [30]. However, scrutiny on Fitzpatrick et al. [20] data reveals that the gonadosomatic index (testes mass/body mass × 100) of pinnipeds is very close to the gonadosomatic index of ungulates from our dataset (mean ± SD gonadosomatic index [range]: 0.06 ± 0.03 [0.02-0.13] in pinnipeds (N = 14) and 0.10 ± 0.19 [0.02-1.23] in ungulates (N = 45); Mann–Whitney-Wilcoxon test: W = 330; p = 0.80). Therefore, the absence of a negative association between weapon length and relative testes mass in ungulates is unlikely to come from a lower post-copulatory sexual selection in this group, which is not surprising since evidence of female multiple mating in ungulates are now compelling (e.g. [4,33]).

Our results further show evidence of a negative association between relative weapon length and sperm length across species of ungulates. When sperm components were analysed separately, this negative association was found only with the length of the sperm tail. In mammals, sperm tail length is correlated with sperm velocity [34], which can potentially increase male fertilization success under competitive conditions [7,8]. Therefore, although an increase in sperm length when the intensity of post-copulatory sexual selection increases is potentially adaptive, such negative covariation between sexual traits such as weapons and microscopic structures like sperm across species is striking and deserves to be studied in other groups before drawing any definitive conclusion. Indeed, this is the first time that a relationship between pre-copulatory male sexually selected traits and sperm characteristics is documented at the inter-specific level although such covariations have been repeatedly investigated at the species level (see [35] for a recent compilation of these studies). For example, in coho salmon (*Onchorhynchus kisutch*) males with more intense spawning colouration, in other words that allocate heavily in secondary sexual characters, had lower sperm velocities than males with less pronounced spawning coloration [36]. Conversely, intra-specific studies have also documented positive relationships between secondary sexual traits and measures of ejaculate quality (e.g. [37]). However, a meta-analysis has recently failed to find a general pattern of covariation between pre- and post-copulatory traits [35], suggesting that species ecological and biological characteristics might play a significant role on the direction and the strength of the covariation.

It is particularly surprising to observe a negative relationship between weapon length and sperm length while no association was found between weapon length and testes mass since testes mass is typically considered by far as the most robust indicator of investment in post-copulatory competition [38]. However, as emphasized by Simmons and Fitzpatrick [20] in their recent review on the evolution of male fertility, caution is required before considering relative testes mass as an absolute proxy of sperm competition level. Indeed, testes can perform functions other than sperm production and allocation in testes mass could be favoured even in the absence of variation in the level of sperm competition [39]. In ungulates, testes mass varies between monogamous and polygynous species [26]. However, following the male mating rate hypotheses [39], polygynous species could have heavier testes than monogamous species as a result of a higher number of females to fertilize even in the absence of variation in the level of sperm competition. On the contrary, whether sperm characteristics respond to post-copulatory competition is still a debated topic, especially when, like in our study, no relationship between sperm dimensions and relative testes mass was found. Even if studies reporting either negative relationships or no influence of sperm competition on sperm/tail length have prevented the emergence of a clear picture [40,41], it appears that the majority of comparative studies performed over the past 20 years have reported positive relationships between the level of sperm competition and total sperm length (see Simmons and Fitzpatrick [20] for a review). We thus cannot rule out that other post-copulatory traits such as sperm dimensions could respond to post-copulatory sexual selection [8].

Finally, preferential investment in pre- or post-copulatory traits could be obscured by variation in the pre- and post-copulatory selective pressures resulting from their mating tactics. Indeed in the moth (*Plodia interpunctella*) mating tactic is associated with differences in ejaculate expenditure. Moth males with female defence mating tactic have larger heads and thoraxes, have smaller testes and produce fewer sperm than males without female defence mating tactic [42]. However, our results do not show distinct pattern of allocation to pre- and post-reproductive traits according to the mating tactics. Indeed, none of the pre- and post-copulatory traits investigated differ between mating tactics nor is the covariation between sperm dimensions (total sperm and tail length) and weapon length affected by mating tactics. Discrepancies between our predictions and our results might have at least two explanations. First, the classification of mating tactics used (female defence versus territory defence) might not reflect adequately the level of pre- and post-copulatory competition in ungulate species. For instance, in the great kudu (*Tragelaphus strepsiceros*), a non-territorial species, the level of pre-copulatory competition might be, contrary to our hypothesis, not so elevated since in this species males fight only occasionally [43]. Conversely, the level of post-copulatory competition can also be high in species commonly classified as female defence mating tactic such as the white-tailed deer (*Odocoileus virginianus)*. Indeed, in this species, DNA microsatellite markers reveal that multiple paternities are widespread within litters [33]. Second, in ungulates, males can show strong plasticity in their reproductive behaviour shifting from one mating tactic to another (see [44] for a review on this topic). In this study, we have used the main mating tactic generally associated with the species, but we cannot exclude that the degree of the occurrence of alternative mating tactics might exert selective pressures. For example, within a given species, individuals adopting a territory defence tactic are predicted to have large weapons and low testes mass while individuals adopting a female defence mating strategy are predicted to have small weapons and high testes mass. These individual variations in mating tactic might obscure the relationships between pre- and post-copulatory traits at the species level. Unfortunately high quality data on reproductive tactics in ungulates are currently not sufficient to obtain a classification of mating tactics that would better reflect the selective pressures exerted on pre- and post-copulatory traits, limiting the conclusions that can be drawn from the absence of distinct pattern of allocation to pre- and post-reproductive traits reported.

## Conclusions

Our results reveal the absence of a negative association between pre-copulatory male sexually selected traits and relative testes mass. Nevertheless, a negative association between pre-copulatory male sexually selected traits and sperm tail length was found. Covariations at the inter-specific level between sexual traits or others are sometimes interpreted as an evolutionary trade-off (e.g. [20]) although such interpretations require extreme caution [18]. Indeed, these conclusions are often based on the assumption that the quantity of resources devoted to the production and maintenance of sexual traits should be the same across species [6,45]. In ungulates, the variance in mass-specific metabolic rate appears to be small compared to other mammalian taxa [30]. Therefore, the amount of resources that males can allocate to sexual traits is likely to be roughly similar between these species although fine scale measurements would be needed before drawing any definitive conclusion on the absence or presence of an evolutionary trade-off. Further studies on the relative costs and benefits associated with investment in different sexual traits in ungulates (see also [20]), through for instance the use of experimental manipulations (e.g. [19]) and mating trials, are now required before drawing any conclusion on the presence of an evolutionary trade-off.

## Methods

### Dataset

Data were collected on adult males of *Bovidae* and *Cervidae* species, the two main families of weaponed *Artiodactyla*. We first conducted two separate literature surveys: one on weapon length (horn length for *Bovidae* and antler length for *Cervidae*). Once the maximum number of data on weapon length was collected, we focused our literature survey on testes mass and sperm length, as well as sperm head length, sperm midpiece length and volume, and sperm tail length, since sperm is a complex cell divided in three main structures. Additionally, following the classification proposed by Bro-Jørgensen [23] and later used by Plard et al. [29], each species was characterized as having either a female defence mating tactic when they guard a female during their receptive period (level 0 in statistical analysis) or a territory defence mating tactic (level 1 in statistical analysis) depending on the males main mating tactic. For territory defence category, both studies [23,29] merge territorial species (where individuals defend large territories with food resources) and lekking species (where individuals defend small territories with no food resources but females) since lekking could be an alternative mating tactic of territorial species. Moreover, no intra-specific differences in weapon size have been found between territorial and lekking populations and thus combining both groups is justified [23,29]. The dataset was then supplemented using information from more specific sources. All data used were checked from the original source and are provided in the Additional file 4: Table S4.

The final dataset contains information on weapon length, body mass, paired testes mass, sperm dimensions and mating tactics for 58 *Artiodactyla* species including 40 species of *Bovidae* and 18 species of *Cervidae.* Since testes mass and all sperm dimensions were not available for all species, sample sizes vary from 31 to 58 species in the statistical analysis.

### Comparative method

Species may share characteristics as a result of a common ancestry. This could create dependency among the data, which potentially compromises statistical tests [46]. On *Artiodactyla*, no consensus phylogeny has been reached yet and we thus used two different phylogenies with topology and branch length (Additional file 5: Figure S1).

The first phylogenetic tree was derived from the phylogenetic supertree of mammals of Bininda-Emonds et al. [31] and is commonly used in inter-specific studies focused on *Artiodactyla*. However, this tree displays an important number of polytomies and we thus repeated our analyses with another phylogenetic tree derived from Agnarsson and May-Collado [32]. This second phylogenetic tree presents no polytomies. Unfortunately, five species from our dataset were absent from this tree: sunda sambar (*Cervus timorensis*), blue wildebeest (*Connochaetes taurinus),* lechwe (*Kobus leche)*, red brocket (*Mazama americana*), and common duiker (*Sylvicapra grimmia*). Consequently, both trees were used to compare consistency between models using a tree containing the whole set of species but containing polytomies [31] and using a tree containing no polytomies but without five species from our dataset [32].

We then used phylogenetic generalized linear models (PGLS). This statistical method estimates an index called λ that indicates whether the phylogeny correctly predicts the patterns of covariance among species on a given trait. λ varies between 0 (complete absence of phylogenetic structure, i.e*.* the phylogenetic structure can be represented by a star phylogeny) and 1 (phylogenetic structure in agreement with a Brownian model of the evolution of the considered traits, i.e*.* the phylogenetic structure can be represented by the previously constructed tree with unmodified topology and branch length) [47]. *λ* is then introduced in the model to control for the phylogenetic effect by multiplying all the off-diagonal values of the variance-covariance matrix extracted, with the R-package ‘ape’ [48], from the constructed phylogenetic tree. Then, the generalized linear model is fitted with this modified variance-covariance matrix [47].

### Statistical analysis

All statistical analyses were conducted with the two phylogenetic trees described above. However, unless otherwise stated, the results were qualitatively identical with these two phylogenetic trees. Therefore, only results from Bininda-Emonds et al. [31] are presented in the manuscript, although those from Agnarsson and May-Collado [32] can be found in Additional file 2: Table S2.

Since weapons differ in structure and growth rate between *Bovidae* and *Cervidae*, we first tested for an effect of the taxonomic family on each studied trait. We thus built PGLS models with each male sexual trait (pre- and post-copulatory) considered as the dependent variable and family (coded as 0 for *Bovidae* and 1 for *Cervidae*) as the independent variable. Since no difference in any of the traits studied could be found between *Bovidae* and *Cervidae* families, both families were considered together in all subsequent analyses (Additional file 3: Table S3).

Due to the role of sperm quantity and quality in fertilization success [7], both traits may have co-evolved leading to dependencies between the associated traits. In order to test the relationship between these traits, we first analysed, using the PGLS regressions, the relationship between relative testes mass and sperm dimensions as well as between sperm dimensions.

Once these analyses were conducted, we proceeded to the test of our two hypotheses. First, to test for a negative association between traits involved in pre- and post-copulatory sexual competition, we constructed a series of PGLS models including weapon length as a dependent variable and either testes mass, total sperm length or any sperm component dimensions as independent variables. To further control for a potential confounding effect of the taxonomic family on the association between pre- and post-copulatory traits, we included an additive or an interactive effect of species family with each post-copulatory traits in the model described above. The absence of an additive or an interactive effects of the family on the relationship between traits (see Additional file 3: Table S3), further confirm that both families can be considered together in the presented analyses. Furthermore, analyses conducted on the species belonging to the *Bovidae* species only (the number of species of *Cervidae* being too small (*N =* 18) for separate analyses) gave qualitatively similar results and are thus not presented.

Second, to test for an effect of the mating tactic on investment in pre- and post-copulatory traits, we also constructed PGLS models with each trait (weapon length, testes mass, total sperm length or each sperm components length) considered as the dependent variables and mating tactics (coded as 0 for female defence mating tactic and coded as 1 for territory defence mating tactic) as the independent variable. We further investigated whether the association between traits involved in pre- and post-copulatory sexual competition could be modulated by the mating tactics. For this purpose, we tested for an interactive effect of the mating tactics with either relative testes mass, total sperm length or any sperm component dimensions on weapon length. Here, again, analyses conducted on the species belonging to the *Bovidae* species only gave qualitatively similar results and are not presented.

Whenever relationships between two traits or more were investigated, all variables were log transformed to linearize the relationship. Log transformed male body mass was included as an independent variable to control for allometric relationship in any of the above models involving weapon length or testes mass, but not in models involving sperm dimensions since preliminary analyses failed to evidence any allometric relationship between sperm dimensions and body mass. Finally, for each model, normality of the residuals was checked using standard diagnostic plots (density plots of the distribution of the residuals and normal Q-Q plots) and the fit to the data was assessed graphically by plotting the fitted values against both the residuals and the observed data.

All statistical analyses were conducted using the packages ‘ape’ , ‘mvtnorm’ , ‘adephylo’ , ‘caper’ and ‘phylobase’ of R version 2.15.0 R [49]. Effect sizes (r) were calculated from the t-values and degrees of freedom from PGLS models [50]. Unless otherwise stated, all tests were two-tailed, the level of significance was set to 0.05, and parameter estimates are given ± SE.

## Competing interests

The authors declare that they have no competing interests.

## Authors’ contributions

Conceived and designed the experiments: AC, JFL and MFR. Analysed the data: MFR and AC. Wrote the paper: MFR, JFL, AC and BC. Collected the data: MFR, SL and AC. All authors read and approved the final manuscript.

## Supplementary Material

Additional file 1: Table S1 Phylogenetically corrected models testing **(a)** the relations between testes mass as dependent variable and the dimensions of the different sperm components (total sperm length, head length, midpiece length, tail length and midpiece volume) across ungulate species considering phylogenetic tree based on Bininda-Emonds et al. [31] and Agnarsson and May-Collado [32]; and **(b)** between the dimensions of the different sperm components (total sperm length, head length, midpiece length, tail length and midpiece volume) across ungulate species obtained when considering the phylogenetic tree based on Bininda-Emonds et al. [31]; and **(c)** Agnarsson and May-Collado [32]. The superscripts following the λ value indicate p-value of likelihood ratio tests against models with λ = 0 (first position) and λ = 1 (second position). Click here for file

Additional file 2: Table S2 Phylogenetically corrected models with Agnarsson and May-Collado [32] phylogeny across ungulates species showing **(a)** the relationships between pre- (weapon length) and post-copulatory traits (testes mass, total sperm length, head length, midpiece length, tail length and midpiece volume); **(b)** differences in the size of pre- and post-copulatory traits between species with different mating tactics; and **(c)** relationships between weapon length and both post-copulatory traits (testes mass, total sperm length, head length, midpiece length, tail length and midpiece volume) and mating tactics. For mating tactics, ‘female defence’ is coded as 0 and ‘territory defence’ is coded as 1. λ represents the index of phylogenetic covariance (see Methods section). The superscripts following the λ value indicate p-value of likelihood ratio tests against models with λ = 0 (first position) and λ = 1 (second position). *variables without log transformation. Click here for file

Additional file 3: Table S3 Phylogenetically corrected models showing no effect of the taxonomic family (*Cervidae* or *Bovidae*) to which a species belongs to on the pre-copulatory traits (weapon length) and the post-copulatory traits (testes mass, total sperm length, head length, midpiece length, tail length and midpiece volume) **(a)** or on the relations between pre-copulatory and post-copulatory traits **(b)**. Only the additive models were presented since no significant interactions were found. The superscripts following the λ value indicate p-value of likelihood ratio tests against models with λ = 0 (first position) and λ = 1 (second position). *means that variables were log transformed. Click here for file

Additional file 4: Table S4 Dataset used in the analyses. Click here for file

Additional file 5: Figure S1 Phylogenetic reconstruction for the 58 ungulate species used in the phylogenetically corrected models. These reconstructions were based on Bininda-Emonds et al. [31]**(a)**; and Agnarsson and May-Collado [32]**(b)**. The five species in bold are missing from the phylogenetic tree derived from Agnarsson and May-Collado [32], which contains 53 species. Click here for file
