# Supplementary material for: Do pre- and post-copulatory sexually selected traits covary in large herbivores?
Source: BMC Evol Biol. 2014 Apr 10;14:79. doi: 10.1186/1471-2148-14-79 (PMC4026391; doi:10.1186/1471-2148-14-79)
Supplement: Additional file 1: Table S1 — Phylogenetically corrected models testing (a) the relations between testes mass as dependent variable and the dimensions of the different sperm components (total sperm length, head length, midpiece length, tail length and midpiece volume) across ungulate species considering phylogenetic tree based on Bininda-Emonds et al. [31] and Agnarsson and May-Collado [32]; and (b) between the dimensions of the different sperm components (total sperm length, head length, midpiece length, tail length and midpiece volume) across ungulate species obtained when considering the phylogenetic tree based on Bininda-Emonds et al. [31]; and (c) Agnarsson and May-Collado [32]. The superscripts following the λ value indicate p-value of likelihood ratio tests against models with λ = 0 (first position) and λ = 1 (second position). [file 1471-2148-14-79-S1.pdf]

Additional file 1: Table S1. Phylogenetically corrected models testing (a) the relations between testes mass as dependent variable and the dimensions of the different sperm components (total sperm length, head length, midpiece length, tail length and midpiece volume) across ungulate species considering phylogenetic tree based on Bininda-Emonds et al. [31] and Agnarsson and May-Collado [32]; and (b) between the dimensions of the different sperm components (total sperm length, head length, midpiece length, tail length and midpiece volume) across ungulate species obtained when considering the phylogenetic tree based on Bininda-Emonds et al. [31]; and (c) Agnarsson and May-Collado [32].

(a)		Phylogeny from Bininda-Emonds et al. [31]					Phylogeny from Agnarsson and May-Collado [32]				
Dependent variable	Independent variables	beta ± SE	t	P	N	λ	beta ± SE	t	P	N	λ
Testes mass	Body mass	0.64 ± 0.11	6.11	< 0.001	41	$< 0.001^{1 / <0.001}$	0.62 ± 0.11	5.73	< 0.001	37	$< 0.001^{1 / <0.001}$
	Total sperm length	-0.05 ± 1.28	-0.04	0.97			1.12 ± 1.46	0.77	0.45		
Testes mass	Body mass	0.62 ± 0.11	5.84	< 0.001	41	$< 0.001^{1 / <0.001}$	0.62 ± 0.11	5..60	< 0.001	37	$< 0.001^{1 / <0.001}$
	Head length	-0.81 ± 0.77	-1.04	0.30			-0.35 ± 0.90	-0.39	0.70		
Testes mass	Body mass	0.65 ± 0.11	5.82	< 0.001	40	$< 0.001^{1 / <0.001}$	0.64 ± 0.12	5.55	< 0.001	36	$< 0.001^{1 / <0.001}$
	Midpiece length	-0.05 ± 0.62	-0.08	0.94			0.26 ± 0.69	0.38	0.71		
Testes mass	Body mass	0.62 ± 0.12	5.27	< 0.001	34	$< 0.001^{1 / <0.001}$	0.63 ± 0.12	5.05	< 0.001	31	$< 0.001^{1 / <0.001}$
	Midpiece volume	-0.13 ± 0.38	-0.33	0.74			-0.18 ± 0.42	-0.44	0.67		
Testes mass	Body mass	0.64 ± 0.11	6.02	< 0.001	41	$< 0.001^{1 / <0.001}$	0.61 ± 0.11	5.43	< 0.001	37	$< 0.001^{1 / <0.001}$
	Tail length	0.03 ± 1.10	0.02	0.98			0.70 ± 1.17	0.60	0.55		

The superscripts following the λ value indicate p-value in likelihood ratio tests against models with λ=0 (first position) and λ=1 (second position).

(b)	Total sperm length	Head length	Midpiece length	Midpiece volume
Head length	**0.49**<br>54 *<0.001*			
Midpiece length	**0.69**<br>53 *<0.001*	**0.26**<br>53 *0.04*		
Midpiece volume	**0.28**<br>47 *0.03*	**0.27**<br>47 *0.04*	**0.23**<br>47 *0.06*	
Tail length	**0.86**<br>54 *<0.001*	**0.20**<br>54 *0.08*	**0.30**<br>53 *0.02*	**0.17**<br>47 *0.12*

Correlation coefficients, their associated sample sizes and p-values are respectively given in bold, roman and italic script.

(c)	Total sperm length	Head length	Midpiece length	Midpiece volume
Head length	**0.38**<br>49 *0.004*			
Midpiece length	**0.65**<br>48 *<0.001*	**0.06**<br>48 *0.02*		
Midpiece volume	**0.25**<br>43 *0.06*	**0.23**<br>43 *0.07*	**0.24**<br>43 *0.07*	
Tail length	**0.88**<br>49 *<0.001*	**0.15**<br>49 *0.16*	**0.18**<br>48 *0.11*	**0.18**<br>43 *0.13*

Correlation coefficients, their associated sample sizes and p-values are respectively given in bold, roman and italic script.
