# Supplementary material for: Do pre- and post-copulatory sexually selected traits covary in large herbivores?
Source: BMC Evol Biol. 2014 Apr 10;14:79. doi: 10.1186/1471-2148-14-79 (PMC4026391; doi:10.1186/1471-2148-14-79)
Supplement: Additional file 2: Table S2 — Phylogenetically corrected models with Agnarsson and May-Collado [32] phylogeny across ungulates species showing (a) the relationships between pre- (weapon length) and post-copulatory traits (testes mass, total sperm length, head length, midpiece length, tail length and midpiece volume); (b) differences in the size of pre- and post-copulatory traits between species with different mating tactics; and (c) relationships between weapon length and both post-copulatory traits (testes mass, total sperm length, head length, midpiece length, tail length and midpiece volume) and mating tactics. For mating tactics, ‘female defence’ is coded as 0 and ‘territory defence’ is coded as 1. λ represents the index of phylogenetic covariance (see Methods section). The superscripts following the λ value indicate p-value of likelihood ratio tests against models with λ = 0 (first position) and λ = 1 (second position). *variables without log transformation. [file 1471-2148-14-79-S2.pdf]

Additional file 2:Table S2. Phylogenetically corrected models with Agnarsson and May-Collado [32] phylogeny across ungulates species showing (a) the relationships between pre- (weapon length) and post-copulatory traits (testes mass, total sperm length, head length, midpiece length, tail length and midpiece volume); (b) differences in the size of pre- and post-copulatory traits between species with different mating tactics; and (c) relationships between weapon length and both post-copulatory traits (testes mass, total sperm length, head length, midpiece length, tail length and midpiece volume) and mating tactics. Mating tactics signify ('female defence' species coded as 0 and 'territory defence' coded as 0 mating tactic). λ represents the index of phylogenetic covariance (see Methods section). The superscripts following the λ value indicate p-value in likelihood ratio tests against models with λ=0 (first position) and λ=1 (second position).*variables without log transformation

(a)	Dependent variables	Independent variables	beta ± SE	*t*	*P*	*N*	df	r	λ
	Weapon length	Body mass	0.47 ± 0.08	5.61	< 0.001	41	38	0.67	$0.95^{1 / 0.45}$
		Testes mass	0.03 ± 0.07	0.46	0.65			0.07	
	Weapon length	Body mass	0.53 ± 0.05	10.10	< 0.001	49	46	0.85	$1.00^{<0.001 / 1}$
		Sperm length	-1.09 ± 0.53	-2.05	0.05			-0.32	
	Weapon length	Body mass	0.54 ± 0.05	9.98	< 0.001	49	46	0.83	$0.99^{<0.001 / 0.56}$
		Head length	-0.14 ± 0.36	-0.38	0.70			-0.06	
	Weapon length	Body mass	0.53 ± 0.06	9.14	< 0.001	48	45	0.81	$0.98^{<0.001 / 0.41}$
		Midpiece length	-0.19 ± 0.33	-0.57	0.57			-0.09	
	Weapon length	Body mass	0.51 ± 0.06	9.06	< 0.001	43	40	0.82	$1.00^{<0.001 / 1}$
		Midpiece volume	0.12 ± 0.08	1.54	0.13			0.24	
	Weapon length	Body mass	0.54 ± 0.05	10.27	< 0.001	49	46	0.83	$1.00^{<0.001 / 1}$
		Tail length	-0.74 ± 0.40	-1.85	0.07			-0.26	

(b) Dependent variables	Independent variables	beta ± SE	*t*	*P*	*N*	df	r	λ
Weapon length	Body mass	0.48 ± 0.07	7.04	< 0.001	53	50	0.71	$0.98^{<0.001 / 0.53}$
	Tactic	-0.27 ± 0.17	-1.60	0.12			-0.22	
Testes mass	Body mass	0.60 ± 0.13	4.67	< 0.001	41	38	0.60	$< 0.001^{1/<0.001}$
	Tactic	-0.01 ± 0.29	-0.05	0.96			-0.01	
Sperm length*	Tactic	-0.95 ± 2.03	-0.47	0.64	49	47	-0.07	$0.85^{1 / 0.01}$
Head length*	Tactic	-0.09 ± 0.42	-0.21	0.84	49	47	-0.03	$0.94^{0.01 / 0.36}$
Midpiece length*	Tactic	-0.77 ± 0.79	-0.99	0.33	48	46	-0.14	$0.97^{0.02 / 0.13}$
Midpiece volume*	Tactic	-0.21 ± 0.41	-0.53	0.60	43	41	-0.08	$< 0.001^{1 / <0.001}$
Tail length*	Tactic	0.20 ± 1.31	0.16	0.88	49	47	0.02	$< 0.001^{1 / <0.001}$

(c) Dependent variables	Independent variables	beta ± SE	*t*	*P*	*N*	df	r	λ
Weapon length	Body mass	0.42 ± 0.09	4.49	< 0.001	41	37	0.59	$< 0.001^{1 / 0.27}$
	Testes mass	-0.05 ± 0.12	-0.44	0.66			-0.07	
	Tactic	-0.71 ± 0.62	-1.15	0.26			-0.19	
	Testes mass x Tactic	0.11 ± 0.15	0.71	0.48			0.12	
Weapon length	Body mass	0.47 ± 0.06	7.78	< 0.001	49	45	0.76	$1.00^{<0.001 / 1}$
	Sperm length	-1.18 ± 0.81	-1.46	0.15			-0.21	
	Tactic	-0.10 ± 4.31	-0.02	0.98			-0.03	
	Sperm length x Tactic	-0.05 ± 1.05	-0.04	0.96			-0.01	
Weapon length	Body mass	0.49 ± 0.06	7.86	< 0.001	49	45	0.76	$0.99^{<0.001 / 0.54}$
	Head length	0.02 ± 0.84	0.03	0.98			0.004	
	Tactic	0.20 ± 1.91	0.11	0.92			0.02	
	Head length x Tactic	-0.22 ± 0.91	-0.24	0.81			-0.4	
Weapon length	Body mass	0.46 ± 0.07	6.83	< 0.001	48	44	0.72	$0.96^{0.001 / 0.16}$
	Midpiece length	-0.15 ± 0.49	-0.31	0.76			-0.05	
	Tactic	1.01 ± 1.61	0.63	0.53			0.09	
	Midpiece length x Tactic	-0.53 ± 0.63	-0.83	0.41			-0.12	
Weapon length	Body mass	0.47 ± 0.07	7.16	< 0.001	43	39	0.75	$1.00^{<0.001 / 1}$
	Midpiece volume	0.17 ± 0.16	1.02	0.32			0.16	
	Tactic	-0.15 ± 0.26	-0.59	0.56			-0.09	
	Midpiece volume x Tactic	-0.07 ± 0.19	-0.37	0.72			-0.06	
Weapon length	Body mass	0.49 ± 0.06	7.98	< 0.001	49	45	0.77	$1.00^{<0.001 / 1}$
	Tail length	-0.82 ± 0.62	-1.31	0.20			-0.19	
	Tactic	-0.74 ± 3.01	-0.25	0.81			-0.04	
	Tail length x Tactic	0.13 ± 0.82	0.16	0.87			0.02	
