# Supplementary material for: Do pre- and post-copulatory sexually selected traits covary in large herbivores?
Source: BMC Evol Biol. 2014 Apr 10;14:79. doi: 10.1186/1471-2148-14-79 (PMC4026391; doi:10.1186/1471-2148-14-79)
Supplement: Additional file 3: Table S3 — Phylogenetically corrected models showing no effect of the taxonomic family (Cervidae or Bovidae) to which a species belongs to on the pre-copulatory traits (weapon length) and the post-copulatory traits (testes mass, total sperm length, head length, midpiece length, tail length and midpiece volume) (a) or on the relations between pre-copulatory and post-copulatory traits (b). Only the additive models were presented since no significant interactions were found. The superscripts following the λ value indicate p-value of likelihood ratio tests against models with λ = 0 (first position) and λ = 1 (second position). *means that variables were log transformed. [file 1471-2148-14-79-S3.pdf]

Additional file 3: Table S3. Phylogenetically corrected models showing no effect of the taxonomic family (*Cervidae* or *Bovidae*) to which a species belongs to neither on the pre-copulatory traits (weapon length) and the post-copulatory traits (testes mass, total sperm length, head length, midpiece length, tail length and midpiece volume) (a) nor on the relations between pre-copulatory and post-copulatory traits (b). Only the additive models were presented since no significant interactions were found. The superscripts following the λ value indicate p-value in likelihood ratio tests against models with λ=0 (first position) and λ=1 (second position).* means that variables were log transformed

(a)		Phylogeny from Bininda-Emonds et al.[31]					Phylogeny from Agnarsson and May-Collado[32]				
Dependent variables	Independent variables	beta ± SE	t	P	N	λ	beta ± SE	t	P	N	λ
Weapon length*	Body mass*	0.57 ± 0.06	9.19	< 0.001	58	$0.98^{<0.001 / 0.34}$	0.54 ± 0.06	9.15	< 0.001	53	$0.98^{<0.001 / 0.55}$
	Family	0.07 ± 0.45	0.16	0.87			0.25 ± 0.49	0.50	0.62		
Testes mass*	Body mass*	0.64 ± 0.10	6.18	< 0.001	45	$< 0.001^{1 / <0.001}$	0.62 ± 0.11	5.65	< 0.001	41	$< 0.001^{1 / <0.001}$
	Family	-0.20 ± 0.26	-0.78	0.44			-0.10 ± 0.28	-0.36	0.72		
Total sperm length	Family	-0.98 ± 4.20	-0.23	0.82	54	$0.67^{0.08 / <0.001}$	-0.96 ± 5.40	-0.18	0.86	49	$0.84^{1 / 0.01}$
Head length	Family	0.53 ± 0.80	0.66	0.51	54	$0.56^{0.02 / <0.001}$	0.43 ± 1.27	0.34	0.74	49	$0.95^{0.04 / 0.36}$
Midpiece length	Family	-0.59 ± 2.08	-0.28	0.78	53	$0.91^{<0.001 / 0.01}$	-0.26 ± 2.43	-0.11	0.91	48	$0.96^{0.002 / 0.11}$
Midpiece volume	Family	-0.34 ± 0.42	-0.81	0.42	47	$< 0.001^{1 / 0}$	-0.22 ± 0.45	-0.47	0.64	43	$< 0.001^{1 / <0.001}$
Tail length	Family	0.57 ± 2.86	0.20	0.84	54	$0.50^{0.39 / <0.001}$	-0.34 ± 1.39	-0.24	0.81	49	$< 0.001^{1 / <0.001}$

(b)		Phylogeny from Bininda-Emonds et al. [31]					Phylogeny from Agnarsson and May-Collado [32]				
Dependent variable	Independent variables	beta ± SE	t	P	N	λ	beta ± SE	t	P	N	λ
Weapon length*	Body mass*	0.48 ± 0.09	5.58	< 0.001	45	$0.61^{0.57 / 0.18}$	0.47 ± 0.08	5.51	< 0.001	41	$0.94^{1 / 0.41}$
	Testes mass*	0.04 ± 0.09	0.50	0.62			0.04 ± 0.07	0.53	0.60		
	Family	0.21 ± 0.32	0.65	0.52			0.37 ± 0.48	0.77	0.45		
Weapon length*	Body mass*	0.56 ± 0.07	9.91	< 0.001	54	$1.00^{<0.001 / 1}$	0.53 ± 0.05	10.00	< 0.001	49	$1.00^{<0.001 / 1}$
	Total sperm length*	-0.95 ± 0.47	-2.02	0.05			-1.09 ± 0.54	-2.03	0.05		
	Family	0.04 ± 0.41	0.10	0.92			0.23 ± 0.43	0.54	0.59		
Weapon length*	Body mass*	0.57 ± 0.06	10.33	< 0.001	54	$0.98^{<0.001 / 0.30}$	0.54 ± 0.05	9.90	< 0.001	49	$0.99^{<0.001 / 0.56}$
	Head length*	-0.04 ± 0.29	-0.13	0.90			-0.15 ± 0.36	-0.40	0.69		
	Family	0.05 ± 0.40	0.12	0.90			0.24 ± 0.44	0.56	0.58		
Weapon length*	Body mass*	0.56 ± 0.06	9.00	< 0.001	53	$0.98^{<0.001 / 0.26}$	0.53 ± 0.06	9.07	< 0.001	48	$0.98^{<0.001 / 0.41}$
	Midpiece length*	-0.11 ± 0.31	-0.34	0.73			-0.19 ± 0.33	-0.56	0.58		
	Family	0.02 ± 0.41	0.06	0.96			0.21 ± 0.44	0.49	0.63		
Weapon length*	Body mass*	0.51 ± 0.06	8.20	< 0.001	47	$0.98^{<0.001 / 0.21}$	0.51 ± 0.06	8.96	< 0.001	43	$1.00^{<0.001 / 1}$
	Midpiece volume*	0.02 ± 0.07	0.33	0.74			0.12 ± 0.08	1.52	0.14		
	Family	-0.17 ± 0.40	-0.43	0.67			0.14 ± 0.45	0.31	0.76		
Weapon length*	Body mass*	0.57 ± 0.05	10.48	< 0.001	54	$1.00^{<0.001 / 1}$	0.54 ± 0.05	10.18	< 0.001	49	$1.00^{<0.001 / 1}$
	Tail length*	-0.80 ± 0.36	-2.23	0.03			-0.75 ± 0.41	-1.84	0.07		
	Family	0.07 ± 0.40	0.18	0.86			0.24 ± 0.43	0.56	0.58		
