# Supplementary material for: Do pre- and post-copulatory sexually selected traits covary in large herbivores?
Source: BMC Evol Biol. 2014 Apr 10;14:79. doi: 10.1186/1471-2148-14-79 (PMC4026391; doi:10.1186/1471-2148-14-79)
Supplement: Additional file 4: Table S4 — Dataset used in the analyses. [file 1471-2148-14-79-S4.pdf]

Additional file 4: Table S4. Dataset used in the analyses.

Family	Species	Male weapon length (cm)	Male body mass (kg)	Total sperm length (µm)*	Head length (µm)	Midpiece length (µm)	Tail length (µm)	Midpiece volume (µm3)	Testes mass (g)	Mating tactic***
Bovidae	*Addax nasomaculatus*	84.5[c]	117.7[c]	59.08	6.58[b]	14.41[b]	38.09[b]	5.23[b]	43.2[a]	F[c]
	Aepyceros melampus	68.4[c]	56.9[c]	46.15	6.52[p]	7.63[p]	32.00[p]	-	28.9[a]	T[c]
	Alcelaphus buselaphus	57.5[j]	152.1[l]	58.15	5.77[b]	10.04[b]	42.34[b]	4.16[b]	107.0[a]	T[j]
	Ammotragus lervia	73.6[c]	111.8[c]	-	-	-	-	-	18.9[a]	F[c]
	Antidorcas marsupialis	26.5[c]	40.7[c]	57.84	6.47[b]	9.99[b]	41.38[b]	3.09[b]	71.0[a]	T[c]
	Antilope cervicapra	57.0[c]	40.2[c]	62.59	9.01[b]	13.14[b]	40.44[b]	4.66[b]	26.0[e]	T[c]
	Bubalus bubalis	105.0[c]	1200.0[c]	60.98	6.71[b]	11.20[b]	43.07[b]	1.93[b]	652.0[p]	F[c]
	Budorcas taxicolor	41.2[c]	282.7[c]	71.71	7.23[b]	15.37[b]	49.11[b]	3.69[b]	93.4[a]	F[c]
	Capra falconeri	108.0[c]	92.7[c]	62.70	7.95[b]	14.19[b]	40.56[b]	3.23[b]	20.9[a]	F[c]
	Capra hircus	105.0[c]	53.0[c]	57.17	7.54[b]	13.21[b]	36.42[b]	2.76[b]	120.0[e]	F[c]
	Capra ibex	90.0[c]	80.5[c]	58.47	7.08[b]	14.38[b]	37.01[b]	2.31[b]	38.3[a]	F[c]
	Capra nubiana	100.0[c]	70.0[c]	64.44	7.84[b]	14.68[b]	41.92[b]	3.90[b]	-	F[c]
	Capra sibirica	115.1[c]	90.0[c]	54.78	7.54[b]	13.15[b]	34.09[b]	2.54[b]	-	F[c]
	Cephalophus monticola	4.5[c]	4.4[c]	77.50	10.43[b]	20.46[b]	46.61[b]	3.34[b]	-	T[c]
	Cephalophus silvicultor	14.0[h]	52.5[l]	60.41	9.72[b]	16.33[b]	34.36[b]	1.54[b]	33.3[a]	T[h]
	Connochaetes gnou	64.0[c]	166.7[c]	62.28	8.04[i]	11.74[i]	42.50[i]	-	137.0[g]	T[c]
	Connochaetes taurinus	68.0[c]	235.3[c]	46.77	5.01[q]	7.86[q]	33.90[q]	-	306.0[g]	T[c]
	Gazella cuvieri	31.0[c]	29.4[c]	70.40	9.19[b]	10.97[b]	50.24[b]	1.87[b]	39.4[a]	T[c]
	Gazella dorcas	31.5[c]	16.3[c]	62.01	6.09[b]	11.91[b]	44.01[b]	1.76[b]	31.5[a]	T[c]
	Gazella granti	65.0[c]	72.1[c]	55.76	8.34[b]	10.43[b]	36.99[b]	0.61[b]	-	T[c]
	Gazella leptoceros	36.0[c]	27.2[c]	65.31	8.80[b]	12.61[b]	43.90[b]	3.85[b]	6.7[a]	T[c]
	Gazella rufifrons	40.4[c]	27.0[c]	64.72	9.02[b]	12.11[b]	43.59[b]	2.93[b]	7.9[a]	T[c]
	Gazella subgutturosa	30.0[c]	27.4[c]	66.93	9.74[b]	18.80[b]	45.39[b]	4.78[b]	8.2[a]	T[c]
	Gazella thomsonii	34.0[c]	22.7[c]	68.68	9.98[b]	11.42[b]	47.28[b]	6.07[b]	-	T[c]
	Hemitragus jemlahicus	29.5[c]	103.3[c]	-	-	-	-	-	26.7[a]	F[c]
	Kobus ellipsiprymnus	75.0[c]	236.8[c]	61.61	6.98[b]	14.31[b]	40.32[b]	2.95[b]	73.7[a]	T[c]
	Kobus kob	54.5[c]	97.5[c]	-	-	-	-	-	41.7[a]	T[c]
	Kobus leche	70.0[c]	104.3[c]	60.04	8.11[b]	10.99[b]	40.94[b]	3.19[b]	67.9[a]	T[c]
	Kobus megaceros	68.7[c]	105.5[c]	57.98	7.99[b]	11.52[b]	38.47[b]	4.11[b]	52.0[a]	T[c]
	Madoqua guentheri	7.5[c]	3.7[c]	51.66	7.14[b]	12.81[b]	31.71[b]	1.81[b]	3.2[a]	T[c]
	Naemorhedus goral	14.5[c]	32.0[c]	-	-	-	-	-	36.5[a]	T[c]
	Neotragus moschatus	9.0[c]	4.8[c]	70.54	9.68[b]	15.63[b]	45.23[b]	2.58[b]	58.9[a]	T[c]
	Oryx gazella	88.3[c]	178.0[c]	60.30	6.10[b]	12.98[b]	41.22[b]	2.08[b]	37.9[a]	F[c]
	Ovis canadensis	90.0[o]	83.4[l]	64.68	7.34[b]	17.50[b]	39.84[b]	4.03[b]	338.0[a]	F[f]
	Pelea capreolus	22.5[c]	24.0[c]	60.20	4.96[b]	11.27[b]	43.97[b]	1.43[b]	-	T[c]
	Sylvicapra grimmia	9.9[c]	18.3[c]	66.94	9.74[b]	17.08[b]	40.12[b]	1.55[b]	21.3[a]	T[c]
	Tragelaphus angasii	65.0[c]	110.2[c]	63.13	8.55[b]	13.52[b]	41.06[b]	2.49[b]	38.3[a]	F[c]
	Tragelaphus eurycerus	80.0[n]	300.0[q]	60.69	7.81[b]	13.15[b]	39.73[b]	2.22[b]	52.9[a]	F[n]
	Tragelaphus spekii	66.0[c]	102.3[c]	58.26	7.74[b]	13.52[b]	37.00[b]	5.42[b]	-	F[c]
	Tragelaphus strepsiceros	120.0[c]	248.0[c]	69.46	7.62[b]	13.98[b]	47.86[b]	4.60[b]	92.0[a]	F[c]
Cervidae	*Alces alces*	144.0[m]	482.5[m]	62.50[d]	8.80[d]	-	54.70[d]	-	106.0[p]	F[m]
	Axis axis	84.5[m]	89.5[m]	66.30	8.90[k]	13.80[k]	43.60[k]	-	29.8[e]	F[m]
	Axis porcinus	39.9[m]	41.0[m]	67.43	8.70[b]	16.91[b]	41.82[b]	2.98[b]	49.5[a]	F[m]
	Cervus albirostris	115.0[m]	204.0[m]	61.06	8.18[b]	12.93[b]	39.95[b]	3.32[b]	116.4[a]	F[m]
	Cervus duvaucelii	81.3[m]	236.0[m]	63.91	9.35[b]	13.19[b]	41.37[b]	2.39[b]	-	F[m]
	Cervus elaphus	93.6[m]	250.0[m]	61.17	8.35[b]**	13.21[b]**	39.61[b]**	3.01[b]**	97.1[a]**	F[m]
	Cervus eldii	97.2[m]	105.0[m]	62.64	8.20[b]	13.24[b]	41.20[b]	1.30[b]	-	F[m]
	Cervus nippon	48.0[m]	52.0[m]	59.82	8.80[b]	11.20[b]	39.82[b]	2.57[b]	-	T[m]
	Cervus timorensis	67.5[m]	95.5[m]	63.76	8.48[b]	11.03[b]	44.25[b]	2.05[b]	27.4[a]	F[m]
	Cervus unicolor	104.9[m]	192.0[m]	61.67	7.78[b]	12.17[b]	41.72[b]	1.77[b]	77.5[a]	T[m]
	Dama dama	61.5[m]	67.0[m]	64.49	7.96[b]	13.64[b]	42.89[b]	5.96[b]	40.0[a]	T[m]
	Elaphurus davidianus	73.7[m]	214.0[m]	58.84	8.58[b]	10.71[b]	39.55[b]	4.87[b]	57.4[a]	F[m]
	Mazama americana	11.5[m]	24.5[m]	56.09	7.38[b]	10.37[b]	38.34[b]	1.42[b]	-	F[m]
	Muntiacus muntjak	14.2[m]	19.0[m]	58.16	6.37[b]	15.83[b]	35.96[b]	2.59[b]	6.5[a]	T[m]
	Muntiacus reevesi	11.4[m]	13.5[m]	59.75	6.79[b]	12.93[b]	40.03[b]	1.20[b]	-	T[m]
	Odocoileus virginianus	65.6[m]	154.5[m]	52.00	10.00[p]	7.00[p]	35.00[p]	-	85.5[p]	F[m]
	Pudu puda	8.5[m]	13.0[m]	57.98	8.83[b]	12.03[b]	37.12[b]	3.38[b]	-	T[m]
	Rangifer tarandus	91.0[m]	106.5[m]	56.50	7.64[p]	10.38[p]	38.48[p]	-	47.7[a]	F[m]

*until otherwise stated the total sperm length is the sum of the length of the three sperm components, namely : head, midpiece and tail.

** average of the measurements made on the three following *Cervus elaphus* subspecies: *Cervus elaphus bactrianus*, *Cervus elaphus barbarus*, *Cervus elaphus macneillii*.

***F: female defence mating tactic, T: Territory defence mating tactic

References

[a] Anderson MJ, Nyholt J, Dixson AE: **Sperm competition affects the structure of the mammalian vas deferens.** *J Zool* 2004, **264:**97-103.

[b] Anderson MJ, Nyholt J, Dixson AF: **Sperm competition and the evolution of sperm midpiece volume in mammals.** *J Zool* 2005, **267:**135-142.

[c] Bro-Jørgensen J: **The intensity of sexual selection predicts weapon size in male bovids.** *Evolution* 2007, **61:**1316-1326.

[d] Cummins JM, Woodall PF: **On mammalian sperm dimensions.** *J Reprod Fertil* 1985,**75:** 153-175.

[e] Freeman S: **The evolution of the scrotum: a new hypothesis.** *J Theor Biol* 1990, **145:**429-445.

[f] Isvaran K: **Variation in male mating behaviour within ungulate populations: patterns and processes.** *Curr Sci* 2005, **89:**1192-1199.

[g] Ginsberg JR, Rubenstein DI: **Sperm competition and variation in zebra mating-behavior.** *Behav Ecol Sociobiol* 1990, **26:**427-434.

[q] Loison A, Gaillard JM, Pelabon C, Yoccoz NG. **What factors shape sexual size dimorphism in ungulates?** *Evol Ecol Res* 1999, **1:**611–633.

[h] Lumpkin S, Kranz KR: ***Cephalophus sylvicultor*.** *Mamm. Species* 1984, **225:**1-7.

[i] Merilan CP, Read BW, Boever WJ, Knox D, Meehan T: **Comparative characteristics of spermatozoa and semen from 6 species of artiodactylids.** *J Zoo Anim Med* 1982, **13:**69-74.

[j] Nowak RM: **Walker's mammals of the world.** Johns Hopkins Univ Press, 1999.

[k] Patterson MC, Stover J, Westrom WK, Schaffer NS: **Comparative dimensional analysis of spermatozoa of 7 species of *Cervidae*.** *J Zoo Anim Med* 1985, 16: 144-146.

[l] Perez-Barberia FJ, Gordon IJ: **Differences in body mass and oral morphology between the sexes in the *Artiodactyla*: Evolutionary relationships with sexual segregation.** *Evol Ecol Res* 2000, 2:667-684.

[m] Plard F, Bonenfant C, Gaillard J-M: **Revisiting the allometry of antlers among deer species: male-male sexual competition as a driver.** *Oikos* 2011, 120:601-606.

[n] Ralls K: ***Tragelaphus eurycerus.*** *Mamm Species* 1978, **111:**1-4.

[o] Shackleton DM: ***Ovis canadiensis*.** *Mamm Species* 1985, **230:**1-9.

[p] Tourmente M, Gomendio M, Roldan ERS: **Sperm competition and the evolution of sperm design in mammals.** *BMC Evol Biol* 2011, **11:**12.
