# Supplementary material for: Do pre- and post-copulatory sexually selected traits covary in large herbivores?
Source: BMC Evol Biol. 2014 Apr 10;14:79. doi: 10.1186/1471-2148-14-79 (PMC4026391; doi:10.1186/1471-2148-14-79)
Supplement: Additional file 5: Figure S1 — Phylogenetic reconstruction for the 58 ungulate species used in the phylogenetically corrected models. These reconstructions were based on Bininda-Emonds et al. [31](a); and Agnarsson and May-Collado [32](b). The five species in bold are missing from the phylogenetic tree derived from Agnarsson and May-Collado [32], which contains 53 species. [file 1471-2148-14-79-S5.pdf]

**Additional file 5: Figure S1.** Phylogenetic reconstruction for the 58 ungulate species used in the phylogenetically corrected models. These reconstructions were based on Bininda-Emonds et al. [31] (a); and Agnarsson and May-Collado [32] (b). The five species in bold are missing from the phylogenetic tree derived from Agnarsson and May-Collado [32], which contains 53 species.

(a)

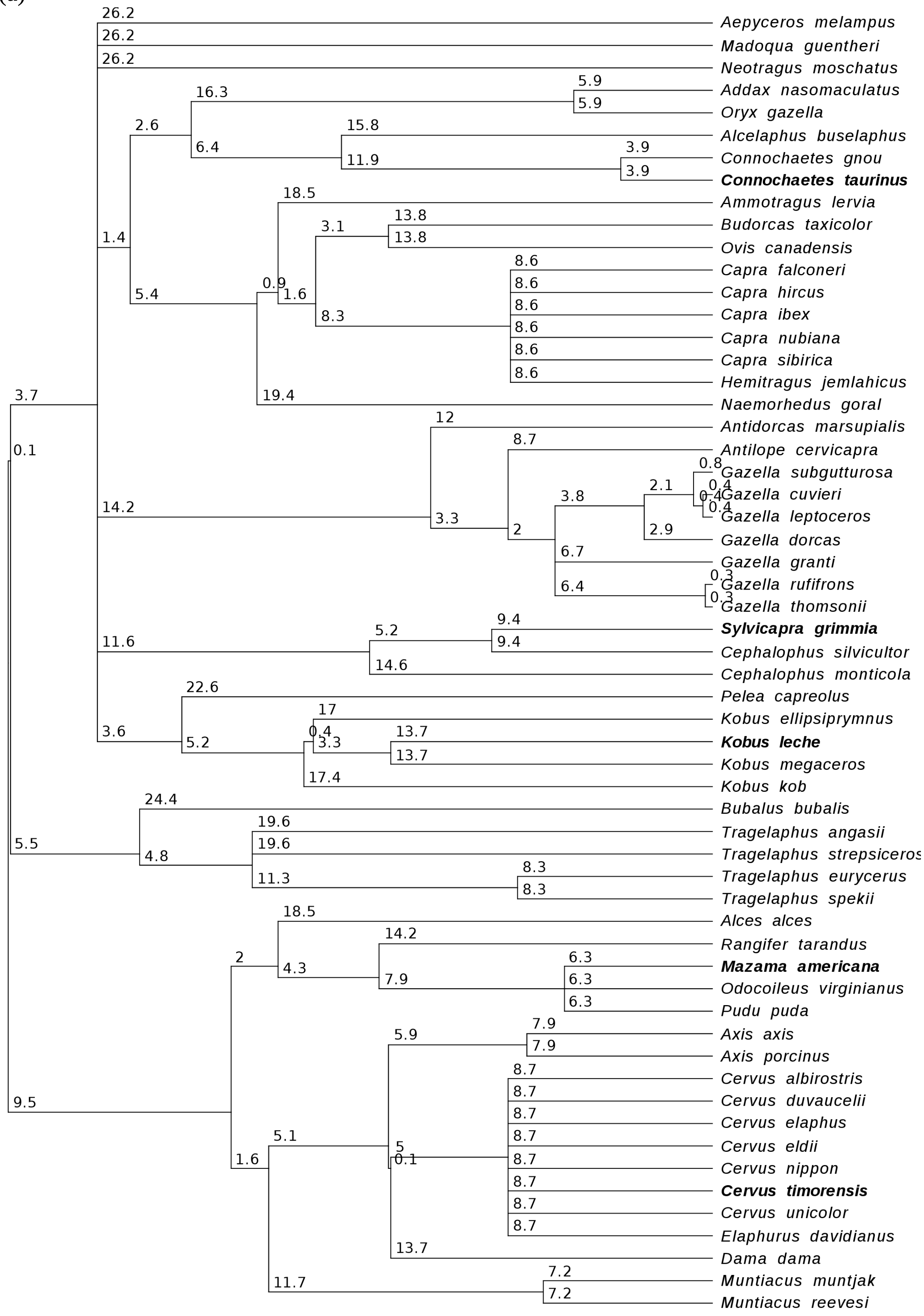

(b)

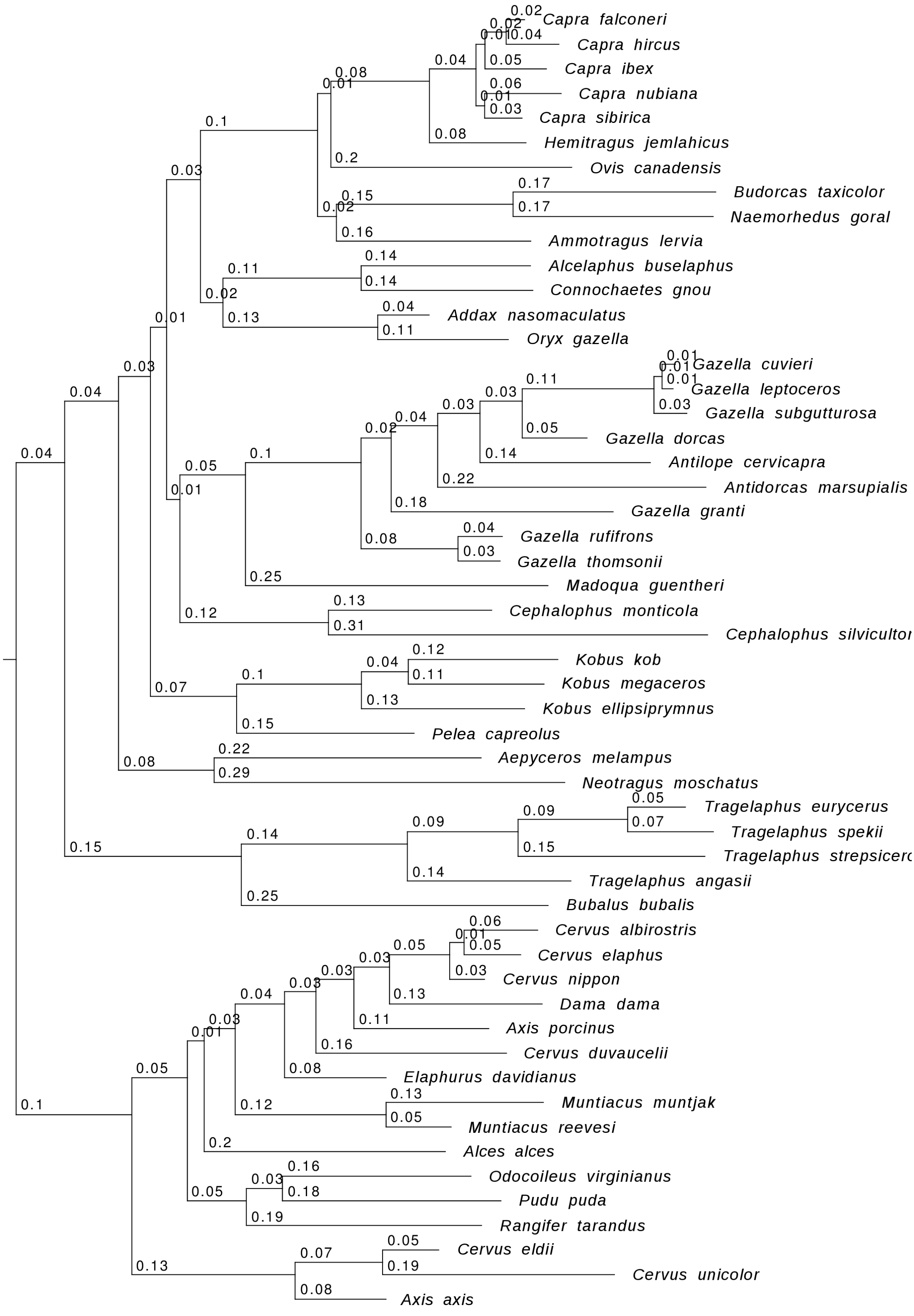
0.02
Capra falconeri
0.02
0.01
0.04
Capra hircus
0.04
0.05
Capra ibex
0.08
0.06
0.01
Capra nubiana
0.01
0.03
Capra sibirica
0.1
0.08
Hemitragus jemlahicus
0.2
Ovis canadensis
0.03
0.17
Budorcas taxicolor
0.15
0.02
0.17
Naemorhedus goral
0.16
Ammotragus lervia
0.11
0.14
Alcelaphus buselaphus
0.02
0.14
Connochaetes gnou
0.04
Addax nasomaculatus
0.01
0.13
0.11
Oryx gazella
0.01
0.01
Gazella cuvieri
0.03
0.01
Gazella leptoceros
0.04
0.11
0.03
Gazella subgutturosa
0.03
0.03
0.04
0.02
0.05
Gazella dorcas
0.04
0.1
0.14
Antilope cervicapra
0.05
0.01
0.22
Antidorcas marsupialis
0.18
Gazella granti
0.04
Gazella rufifrons
0.08
0.03
Gazella thomsonii
0.25
Madoqua guentheri
0.13
Cephalophus monticola
0.12
0.31
Cephalophus silvicultor
0.12
Kobus kob
0.04
0.1
0.11
Kobus megaceros
0.07
0.13
Kobus ellipsiprymnus
0.15
Pelea capreolus
0.22
Aepyceros melampus
0.08
0.29
Neotragus moschatus
0.05
Tragelaphus eurycerus
0.09
0.07
Tragelaphus spekii
0.14
0.09
0.15
0.15
Tragelaphus strepsicero
0.14
Tragelaphus angasii
0.25
Bubalus bubalis
0.06
Cervus albirostris
0.01
0.05
0.05
Cervus elaphus
0.03
0.03
0.03
Cervus nippon
0.04
0.03
0.13
Dama dama
0.03
0.01
0.11
Axis porcinus
0.16
Cervus duvaucelii
0.05
0.08
Elaphurus davidianus
0.13
Muntiacus muntjak
0.1
0.12
0.05
Muntiacus reevesi
0.2
Alces alces
0.16
Odocoileus virginianus
0.03
0.05
0.18
Pudu puda
0.19
Rangifer tarandus
0.05
Cervus eldii
0.07
0.19
Cervus unicolor
0.13
0.08
Axis axis
